# Probiotic properties of *Lactobacillus rhamnosus* GL68 with cholesterol-lowering and antioxidant activities *in vitro* and its effects on SD rats fed with a high-fat diet

**DOI:** 10.3389/fmicb.2026.1830530

**Published:** 2026-06-04

**Authors:** Yurong Gao, Dapeng Li

**Affiliations:** 1 School of Biological and Environmental Engineering, Chaohu University, Chaohu Economic Development Zone, Hefei, China; 2 Chaohu Regional Collaborative Technology Service Center for Rural Revitalization, Chaohu Economic Development Zone, Hefei, China

**Keywords:** antioxidant, cholesterol-lowering, gut microbiota, *Lactobacillus rhamnosus*, lipid metabolic disorders

## Abstract

**Introduction:**

Some probiotic microorganisms have been proven to offer several health benefits. However, there are few reports on lactic acid bacteria with cholesterol-lowering and antioxidant activities *in vitro* and on their probiotic effects on hosts. This study screened and identified probiotics with cholesterol-lowering activity *in vitro* and researched their antioxidant activity and probiotic property *in vitro*, along with their beneficial effects on high-fat diet-induced rats *in vivo*.

**Methods:**

The cholesterol-lowering rate in the cell-free supernatant was used to evaluate the cholesterol-lowering activity of isolates. Tolerance to acid and bile salt and antibacterial activity were assessed to investigate their potential probiotic characteristics, and antibiotic susceptibility was evaluated. DPPH and hydroxyl radical scavenging rate of cell-free supernatant was used to evaluate the antioxidant activity *in vitro*. Body mass, Lee’s index, liver index, blood lipid, liver enzymes levels, histological analysis of liver and pancreas, gut microbiota structure, and short-chain fatty acids were used to evaluate the beneficial effects on high-fat diet-induced rats.

**Results:**

A strain with 71.2 ± 0.9% cholesterol-lowering rate was screened and identified as *Lactobacillus rhamnosus* GL68, and its acid and bile salt tolerance were, respectively, 92.1 ± 4.3% and 88.3 ± 3.2%, and it has antibiotic susceptibility. DPPH and hydroxyl radical scavenging rates were 84.6 ± 1.3% and 79.3 ± 1.1%, respectively. Administering *L. rhamnosus* GL68 to the high-fat diet group significantly reduced weight gain, Lee’s index, and liver index by 22.01, 17.71, and 19.35%, respectively. Additionally, it downregulated total cholesterol, total triglycerides, low-density lipoprotein cholesterol, alanine aminotransferase, and aspartate aminotransferase levels by 29.88, 52.89, 27.51, 26.10, and 26.27%, respectively. Furthermore, it raised high-density lipoprotein cholesterol levels by 20.03%. Administering *L. rhamnosus* GL68 upregulated liver superoxide dismutase and glutathione peroxidase levels by 41.39 and 71.02%, respectively. It downregulated liver malondialdehyde levels by 37.82%. Administering *L. rhamnosus* GL68 ameliorated hepatic inflammation and ballooning degeneration, glandular atrophy, and vesicular degeneration. It also reshaped gut microbiota at the genus level, and showed a positive correlation with short-chain fatty acids in the feces of high-fat diet rats.

**Discussion:**

*L. rhamnosus* GL68 with cholesterol-lowering and antioxidant activity *in vitro* can be used as a potential probiotic to administer high-fat diet-induced lipid metabolism disorder and gut microbiota disorders, and alleviate liver and pancreatic damage.

## 1 Introduction

Lactic acid bacteria (LAB) are generally regarded as probiotics that could exert several beneficial effects on hosts, including resistance to the acidic environment and bile salts in the gastrointestinal tract, maintenance of a balanced intestinal flora, and alleviation of lactose intolerance (Demir, 2020; Aziz et al., 2022; Oak and Jha, 2019). Common sources of LAB are fermented foods, and some LAB strains with potential probiotic effects have been isolated from fermented foods (Gao and Li, 2018; Zhao et al., 2022; Joung et al., 2024; Guney et al., 2025). Research continues to explore the diverse applications of probiotics in addressing conditions such as irritable bowel syndrome (Xie et al., 2011; Aziz et al., 2022) and even systemic health issues (Bubnov et al., 2017; Zhao et al., 2022), highlighting their potential as a complementary approach to conventional medical treatments.

Cholesterol is a biomolecule that is essential for the formation of cell membranes and the synthesis of vitamins and hormones in the mammalian body (Puttarat et al., 2023). However, elevated cholesterol levels, particularly LDL cholesterol, are a major risk factor for atherosclerosis, a chronic progressive disease characterized by arterial narrowing and occlusion that contributes substantially to morbidity and mortality (Xu et al., 2016). To reduce the risk of coronary artery disease, cholesterol-lowering drugs have been widely used in clinical practice; however, these agents may be associated with adverse effects and high costs (Ference et al., 2017). Bubnov et al. (2017) and Zhao et al. (2022) reported beneficial effects of probiotic interventions in obesity- and metabolic syndrome-related settings (Bubnov et al., 2017; Zhao et al., 2022). In contrast, probiotic interventions have not consistently demonstrated significant beneficial effects in systemic conditions such as type 2 diabetes (Meliha et al., 2026). Taken together, these findings suggest that the metabolic efficacy of probiotics in chronic disorders may vary based on the specific probiotic strain or formulation, intervention duration, and disease context. Therefore, it is imperative to further explore the potential of using probiotics to reduce serum cholesterol levels and alleviate metabolic disorders caused by a high-fat diet.

This study aimed to screen and identify probiotics with cholesterol-lowering ability from Chinese fermented cabbage, to assess their potential probiotic characteristics and antioxidant activity *in vitro*, and to evaluate their beneficial effects on high-fat diet-induced rats.

## 2 Materials and methods

### 2.1 Screening of LAB with cholesterol-lowering activity *in vitro* and evaluating acid and bile salt tolerance

Serial 10-fold gradient dilutions of fermented cabbage juice samples were spread on MRS agar, containing 5 g/L of calcium carbonate, and then cultivated at 37 °C for 36 h. Colonies that formed distinct zones of inhibition were randomly selected and purified from the same plates. Then, these isolates were incubated in MRS broth at 37 °C for 24 h, and 2% of the culture was transferred to MRS broth containing cholesterol (100.0 μg/mL; Klamar, Shanghai Spectrum Biotechnology Co., Ltd., China). MRS broth containing the same cholesterol levels but without the strain culture was used as the control. After incubation at 37 °C for 72 h, the supernatant was collected by centrifugation, and cholesterol content was determined using the o-phthalaldehyde method (Gao and Li, 2018). The cholesterol-lowering rate was calculated as follows: cholesterol-lowering rate (%) = (1-C/C_0_) × 100, where C is the cholesterol concentration in the cell-free supernatant (μg/mL) and C_0_ is the cholesterol concentration in the control (μg/mL).

The tolerance to acid and bile salt was evaluated according to the method followed by Gao and Li (2018). The isolates were cultivated in MRS broth at 37 °C for 12 h; the cells were collected by centrifugation and washed third using sterile saline (0.85%) and then resuspended in the same solution adjusted to pH 2.0 with 2.0 mol/L of HCl supplemented with 3 mg/mL of pepsin (Klamar, Shanghai Spectrum Biotechnology Co., Ltd., China) for acid tolerance and in the same solution adjusted to pH 8.0 with 1.0 mol/L NaOH supplemented with 0.3% of bile salt (Klamar, Shanghai Spectrum Biotechnology Co., Ltd., China) and 3 mg/mL of pancreatin (Sigma, Spectrum Biotechnology Co., Ltd., China). After cultivation at 37 °C for 0 and 4 h, the number of viable colonies was determined on MRS agar plates. The survival rate was computed as follows: survival rate (%) = (C/C_0_) × 100%, where C_0_ is the alive colony at 0 h (CFU/mL), and C is the alive colony at 4 h (CFU/mL).

### 2.2 Determination of antibacterial activities

The antibacterial activities of three isolates against six pathogens, including *Staphylococcus aureus* CICC22942 (China Center of Industrial Culture Collection, Beijing, China), *Escherichia coli* CICC25267, *Salmonella enterica* CICC21506, *Listeria monocytogenes* CICC23929, *Vibrio parahaemolyticus* CICC21617, and *Shigella flexneri* CICC21534, were evaluated using agar well diffusion assay (Zhang et al., 2017). These pathogens were grown in nutrient broth (Bawei Biotechnology Limited Liability Company, Guangdong, China) at 37 °C, 100 r/min for 12 h. Then, the culture was diluted to 10^6^ CFU/mL using sterile normal saline, inoculated into nutrient broth soft agar (0.8% agar, 50 °C), and poured on the surface of nutrient broth agar with Oxford cups. After solidification, the Oxford cups were removed, and the wells formed were poured into 100 μL of the cell-free supernatant of three strains. The wells filled with MRS broth were used as controls. After incubation for 24 h at 37 °C, the inhibition diameter was measured with a Vernier caliper.

### 2.3 Evaluation of antioxidant ability *in vitro*

The DPPH radical scavenging ability of isolates was evaluated according to Lin and Chang (2000). Briefly, 1 mL of the cell-free supernatant of fermenting liquor from *L. rhamnosus* GL68 was admixed with 1 mL of 1,1-diphenyl-2-picrylhydrazyl solution (DPPH, 2 × 10^−4^ mol/L) and then placed in darkness for 30 min. A measure of 1 mL of MRS broth was used as the experimental control group. A measure of 1 mL of anhydrous ethanol was used as a blank control group. After incubation, the absorbance at 517 nm was determined, and the scavenging percentage was computed as follows: DPPH scavenging percentage (%) = [1 − (A_Sample_ − A_Control_)/A_Blank_] × 100%.

Hydroxyl radical scavenging ability in the cell-free supernatants of the isolates was calculated according to Peng et al. (2024). The reaction system is prepared by mixing the following components: 2.0 mL of supernatant, 1.0 mL of 5.0 mmol/L of FeSO_4_ aqueous solution, 1 mL of 5 mmol/L of salicylic acid–ethanol solution, and 1.0 mL of 3.0 mmol/L of H_2_O_2_ aqueous solution. After incubation at 37 °C for 15 min, the mixture was centrifuged (4,000 × g, 10 min), and the absorbance of the supernatant at 510 nm was measured. The mixture without H_2_O_2_ was used as the control, and distilled water as the blank control. Hydroxyl scavenging rate was evaluated as follows: hydroxyl scavenging percentage (%) = [1 − (A_Sample_ − A_Control_)/A_Blank_] × 100%.

### 2.4 Molecular identification

Molecular identification of the selected strains was performed using partial 16S rRNA gene sequences according to Gao and Li (2018). After purification using the Agarose Gel DNA Purification Kit (Takara Bio., Dalian, China), the PCR products were sequenced and compared with sequences in the NCBI databases.^1^ The obtained raw sequences were trimmed and compared using MEGA 7 version 7.0.26, with phylogenetic relationship trees (1,000 copies) constructed using sequences similar to those retrieved from the database as references, using the Neighbor-Joining method.

### 2.5 Antibiotic susceptibility of *L. rhamnosus* GL68

Antibiotic susceptibility of the *L. rhamnosus* GL68 was tested using the disk diffusion method (Anandharaj and Sivasankari, 2014). A measure of 0.1 mL of culture overnight (10^7^ CFU/mL) was inoculated on the surface of MRS agar, and the disk containing 10 μg of ampicillin, 30 μg of cefotaxime, 30 μg of chloramphenicol, 10 μg of streptomycin, 10 μg of gentamicin, 10 μg of amoxicillin, 5 μg of ciprofloxacin, and 23.75 μg of sulfamethoxazole standard antibiotics were placed onto the agar surface. After incubating anaerobically at 37 °C for 24 h, the inhibition zone diameter was measured using a Vernier caliper, and the results were analyzed according to the Standards methods (CLSI, 2017).

### 2.6 Feed for Sprague–Dawley rats

Thirty Sprague–Dawley (SD) male rats (130–150 g) were obtained from Spec Bio Biotechnology Co., Ltd. (Production License No.: SCXK (Jing) 2019–0010). All experimental animals were fed adaptively for 7 days in a barrier system at 22–25 °C and 70% relative humidity, and could drink and eat ad libitum. After adaptation, 10 rats were fed standard chow (Jiangsu Collaborative Pharmaceutical Biotechnology Co., Ltd., Jiangsu, China) and served as the control group. The other 20 rats received high-fat chow, supplemented with 20% sucrose, 15% lard, 1.2% cholesterol, and 0.2% sodium cholate. After feeding for 2 weeks, the blood of the inner canthus of the eye was collected. After centrifugation at 4 °C at 4000 × g for 15 min, TC, TG, HDL-C, and LDL-C in serum were analyzed using an Autokinetic Biochemistry Analyzer (Hitachi H-T Co., Japan). TC, TG, and LDL-C values in 20 rats receiving high-fat chow were remarkably higher than those in the control group (control). Subsequently, 20 rats were divided into two groups. There were no significant differences between the two groups in TC, TG, HDL-C, or LDL-C. They were designated as the high-fat diet group (HFD) and the *L. rhamnosus* GL68 group (GL68), respectively. The GL68 group received 2 mL of a culture of *L. rhamnosus* GL68 (10^8^ CFU/mL) intragastrically each day for 4 weeks. Control and HFD groups received 2 mL of sterile normal saline intragastrically each day for 4 weeks.

### 2.7 Determination of body mass, Lee’s index, and liver index

The body mass and chow consumption of each rat were recorded daily. The Lee’s index was determined as follows: Lee’s index = the third root of final mass (g)/nasoanal length (cm). After the completion of the experimental period, animals were euthanized by a single intraperitoneal anesthetic dosage of pentobarbital sodium (50 mg/kg for injection). After the rats were euthanized, the livers were removed, and their liver mass was recorded. The liver index was determined as follows: Liver index = (liver mass/ final body mass) × 100.

### 2.8 Determination of blood lipid, serum ALT, and AST levels

After 4 weeks of feeding, the rats were fasted overnight, and blood in the eye canthus internal was collected. After centrifugation at 4 °C and 4,000 × g for 15 min, TC, TG, HDL-C, LDL-C, ALT, and AST in serum were determined by a Hitachi 7,180 automatic biochemical analyzer (Hitachi High-Technologies Corporation, Japan).

### 2.9 Determination of liver MDA, SOD, and GSH-Px levels in SD rats

The total protein of liver tissue was determined according to the method of Wang et al. (2022). The total protein was extracted using normal saline at 4 °C for 60 min. After centrifugation at 3,000 × g for 15 min, the supernatant was collected, mixed with trichloroacetic acid (1:9 v/v), and precipitated in an ice–water mixture for half an hour. The precipitated protein was then collected by centrifugation (10,000 × g, 5 min), resuspended in 0.1 mol/L of NaOH solution (1:1 v/v), and mixed uniformly. The protein was analyzed by the total protein assay kit (Beijing Bio-Lab Technology Co., Ltd., Beijing, China, Cat: SNM078-VJD). After liver tissue homogeneity with 1:9 normal saline for 20 min and centrifugation (3,000 × g, 15 min), MDA, SOD, and GSH-Px levels of the supernatant were determined by the kits (Jiancheng Bio. Inst. Nanjing, China, Cat: A003-1-1; A001-2-2; A005-1-1).

### 2.10 Histological analysis of liver and pancreas

Ten male rats per group, three sections per rat, and six microscopic fields were examined per section. The partial tissue samples from liver and pancreas were rinsed using physiological saline, fixed in 4% neutral alcohol for 2 days, and then soaked in water for 12 h. Subsequently, the tissues underwent dehydration, clearing, waxing, and embedding to prepare 4-μm paraffin sections. After Hematoxylin and Eosin (HE) staining, the tissue morphology is observed under an optical microscope (Olympus CX23; Olympus, Tokyo, Japan), photographed, and evaluated pathologically. If no pathological changes are observed in the tissue, it is recorded as “normal.” Based on the location (differing regenerative capacity and physiological importance), extent, and severity, lesions are categorized into four grades: mild is recorded as “grade I,” moderate as “grade II,” moderately severe as “grade III,” and severe as “grade IV.”

### 2.11 Analysis of gut microbiota structure and short-chain fatty acids of SD rats

Feces were obtained from SD rats under an aseptic environment 1 day before euthanasia, rapidly frozen in liquid nitrogen to maintain sample integrity, and deposited at −80 °C. Microbial genome DNA of fecal samples was withdrawn using the E. Z. N. A.^®^ soil DNA Kit, and the primer pairs 341F 5′- CCTACGGGNGGCWGCAG −3′ and 806R 5’-GGACTACHVGGGTWTCTAAT-3′ were applied to expand the high-variability region V3-V4 of 16S rRNA gene. Amplicons were extracted and determined by Qubit®3.0 (Life Technologies). The reads were truncated at any site with an average quality score <20 using PRINSEQ, and truncated reads with an N length of 5% of the total sequence length were discarded. Reads that could not be assembled were discarded. The analysis was conducted at the OTU level. This sequence was aligned and analyzed using UCLUST software with a 90% confidence threshold. Finally, the sequences were treated using the MegaSense platform.^2^

The short-chain fatty acids (SCFAs) in fecal samples were analyzed according to Ji et al. (2025). Fecal sample (0.1 g) was blended with 1 mL of water and glass beads, homogenized for 3 min, ultrasonicated for 10 min, and finally centrifuged for 10 min at 8000 × g. After extraction for 15 min with 0.5 mL of methyl tert-butyl ether, the sample was centrifuged (10 °C, 8000 r/min, 15 min). Finally, the supernatant was analyzed GC–MS analysis by a GC–MS/MS-001 Gas Chromatography–Mass Spectrometry Tandem Instrument (Shimadzu, Japan). A column Agilent DB-WAX (30 m × 0.25 mm ID×0.25 μm) and helium carrier at 1 mL/min were used for GC analysis. A splitting mode with a split ratio of 5:1 and an injection temperature of 230 °C was adopted, and 1 μL of sample was injected. The mass spectrometry analysis parameters are as follows: electron impact ion source, source temperature 230 °C, joint temperature 240 °C, and solvent delay 6 min. Using a single-ion detection method, the electron energy is 70 eV.

### 2.12 Statistical analysis

All tests were carried out in triplicate and in duplicate. All dates were analyzed using SPSS 17.0 software (IBM, Armonk, NY, USA) with single-factor analysis of variables, Tukey’s post-hoc test. The significant differences were set at a *p*-value of <0.05.

## 3 Results and discussion

### 3.1 Screening of LAB with cholesterol-lowering ability *in vitro* and their acid and bile salt tolerance

A total of 120 LAB isolates were selected from eight fermentation cabbage samples, and their cholesterol-lowering rates *in vitro* were evaluated. Three strains whose cholesterol-lowering ability was more than 50% were selected. The cholesterol-lowering rate and the survival rate under acid and bile salt are listed in Table 1. The cholesterol concentration in the control (containing 100.0 μg/ mL of cholesterol without the inoculated strain) was 97.2 μg/mL, indicating a 97.2% cholesterol recovery. Among the three selected strains, the cholesterol-lowering rates of the GL68 and GL94 isolates were more than 60%. The acid tolerance of GL68 isolate was insignificantly lower than GL94 (*p*>*0.05*) and significantly higher than GL23 (*p < 0.05*). The bile salt tolerance of GL68 isolate was the highest among these three isolates (88.3 ± 3.2%).

**Table 1:** Cholesterol-lowering rate and survival rate under acid and bile salt of selected isolates.

Stain	Cholesterol-lowering rate (%)	Survival rate under acid (%)	Survival rate under bile salt (%)
GL23	55.3 ± 1.2^c^	82.2 ± 3.6^b^	68.2 ± 1.8^c^
GL68	71.2 ± 0.9^a^	92.1 ± 4.3^a^	88.3 ± 3.2^a^
GL94	62.6 ± 1.7^b^	92.7 ± 2.9^a^	76.1 ± 3.7^b^

^a-c^ Letters indicated that means in the same column were significantly different (*p* **<** 0.05).

Recent studies have suggested that probiotics may offer an economical, non-invasive method with minimal or no delecterious effects to abate dangerous factors for cardiovascular disease, such as TC and LDL (Vourakis et al., 2021; Puttarat et al., 2023). Several LAB such as *L. plantarum* YS5 (Nami et al., 2019), *L. plantarum* E680 (Zheng et al., 2020), *L. plantarum* S9 (Zhao et al., 2022), *L. acidophilus* CL1285 (Frappier et al., 2022), and *Enterococcus durans* NPL 1334 (Rashid et al., 2025) have been reported to exhibit the cholesterol-lowering ability *in vitro*. Cholesterol-reducing rates for most LAB were lower than 70%, indicating that the GL68 isolate exhibited more vigorous cholesterol-lowering activity. To exert beneficial influences on the parasitifer, the capacity to resist an acidic environment and bile salts in the gastrointestinal tract was an essential characteristic as a potential probiotic (Guney et al., 2025). The acid and bile salt resistance of GL68 isolate were 92.1 ± 4.3% and 88.3 ± 3.2%, respectively, and this indicates that there are quite a number of active cells of GL68 isolate remaining after it crosses the gastrointestinal barrier.

### 3.2 Antibacterial activity of selected isolates

As shown in Table 2, the antibacterial activities of the three isolates against the six selected pathogens differed significantly. Both GL23 and GL68 isolates inhibited the selected six pathogens, and the antibacterial activity of the GL68 isolate was considerably stronger than that of the GL23 isolate. However, the GL94 isolate inhibited only the two Gram-positive pathogens and not the four Gram-negative pathogens. As a potential probiotic, antibacterial activity is another crucial characteristic (Zheng et al., 2020). This broad-spectrum antibacterial activity enables the GL68 isolate to inhibit harmful microorganisms in the intestinal tract, thereby effectively modulating the intestinal microbiota.

**Table 2:** Antibacterial activities of three isolates against six selected pathogens.

Isolates	Diameter of inhibition zone (mm)					
	*S. aureus*	*L. monocytogenes*	*S. enterica*	*E. coli*	*V. parahaemolyticus*	*S. flexneri*
GL23	18.17 ± 0.33^c^	21.15 ± 0.47^c^	13.15 ± 0.64^b^	16.74 ± 0.85^b^	12.18 ± 0.34^b^	13.53 ± 0.83^b^
GL68	22.24 ± 0.68^a^	23.74 ± 1.32^a^	17.15 ± 0.72^a^	18.15 ± 0.47^a^	15.42 ± 0.55^a^	19.53 ± 0.83^a^
GL94	21.65 ± 0.40^b^	22.34 ± 0.79^b^	-	-	-	-

^a-c^Letters indicated that means in the same column are significantly different (*p* < 0.05).

### 3.3 Antioxidant activity *in vitro* of selected isolates

DPPH radical scavenging rate of cell-free supernatant of GL23, GL68, and GL94 isolate were 35.1 ± 0.7%, 84.6 ± 1.3%, and 88.3 ± 1.2%, respectively. In addition, the hydroxyl radical scavenging ability of the cell-free supernatants of GL23, GL68, and GL94 isolates was 64.4 ± 0.9%, 79.3 ± 1.1%, and52.7 ± 0.8%, respectively. The GL94 isolate showed the highest DPPH radical scavenging rate; the GL68 isolate was next, and the GL23 isolate was the lowest. Moreover, the GL68 isolate exhibited the highest hydroxyl radical scavenging rate, followed by the GL23 isolate; the GL94 isolate the lowest. A careful consideration of the free radical scavenging capacities indicates that the GL68 strain exhibited the best antioxidant capacity *in vitro* among the three strains, with DPPH and hydroxyl radical scavenging ability of cell-free supernatant being 84.6 ± 1.3% and 79.3 ± 1.1%, respectively. Several previous reports have indicated that probiotic strains such as *L. casei* (Lee et al., 2005; Qian et al., 2023) and *L. reuteri* (Song et al., 2023) exhibited radical scavenging activity. In addition, studies indicated that the radical scavenging activity of probiotics exhibited high strain-related variability (Amaretti et al., 2013).

### 3.4 Molecular identification

GL68 isolate presented comparatively higher cholesterol-reducing capacity, bile salt tolerance, antibacterial activity, and hydroxyl radical scavenging rate compared to GL23 isolate and GL94 isolate, as well as significantly higher acid tolerance and DPPH radical scavenging rate than GL23 isolate. Therefore, the isolated GL68 was selected for strain molecular identification and subsequent animal experiments.

The nucleotide sequence of a 1502 bp fragment was amplified from the 16S rRNA genome of isolate GL68 and compared with sequences in NCBI; the results suggested that the GL68 strain exhibited the highest homology percentage (100%) with *Lactobacillus rhamnosus* ATCC 53103 (AY370682.1). Therefore, the GL68 isolate was identified as *L. rhamnosus*, and the GenBank access number is PX671531.

### 3.5 Antibiotic susceptibility of *L. rhamnosus* GL68

As shown in Table 3, *L. rhamnosus* GL68 showed sensitivity to ampicillin, cephalothin, streptomycin, gentamicin, amoxicillin, and sulfamethoxazole, intermediate sensitivity to chloramphenicol and ciprofloxacin. Since microorganisms’ resistance to antibiotics has become a public health issue, there is concern that probiotics may exacerbate this problem due to their intrinsic tolerance to certain clinically antibiotics (Imperial and Ibana, 2016; Zheng et al., 2020). This antibiotic susceptibility results suggested that *L. rhamnosus* GL68 could not serve as a potential conduit for the transfer of antibiotic resistance through food. The antibiotic susceptibility results suggested that lactic acid bacteria have good host safety.

**Table 3:** Antibiotic susceptibility of *L. rhamnosus* GL68.

Antibiotic	Inhibition diameter (mm)	Susceptibility*
Ampicillin	25.34 ± 0.36	S
Cephalothin	24.71 ± 0.24	S
Chloramphenicol	16.27 ± 0.18	I
Streptomycin	26.34 ± 0.53	S
Gentamicin	18.23 ± 0.39	S
Amoxicillin	21.46 ± 0.18	S
Ciprofloxacin	18.35 ± 0.41	I
Sulfamethoxazole	17.23 ± 0.30	S

* Alphabet indicated that the isolate was resistant (R), sensitive (S), or intermediate (I) to the antibiotics.

### 3.6 Effect of *L. rhamnosus* GL68 on body mass, Lee’s index, and liver index of HFD-induced rats

**Figure 1:**
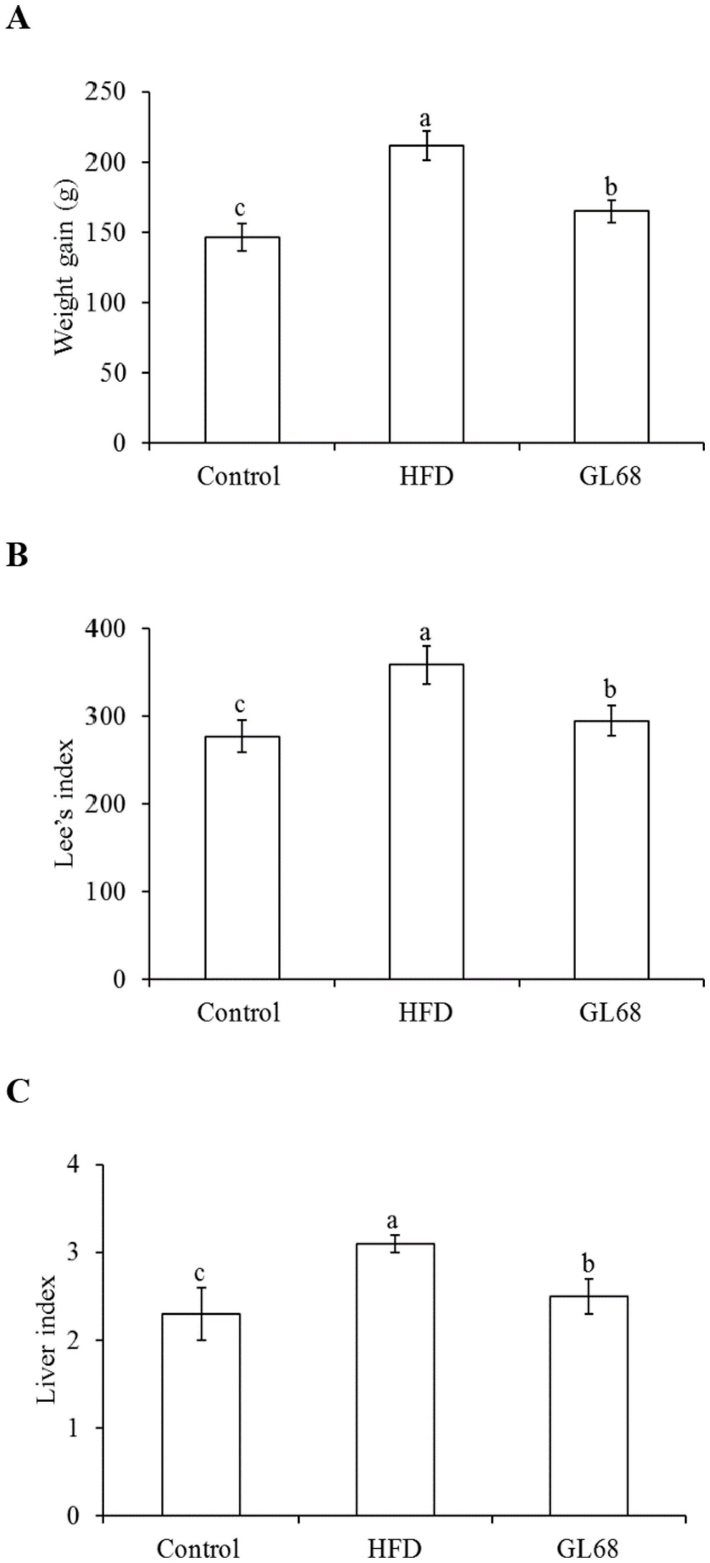
Effects of *L. rhamnosus* GL68 on weight gain **(A)**, Lee’s index **(B)**, and liver index **(C)** of HFD SD rats.

The mass gain, Lee’s index, and liver index in HFD group significantly raised in comparison with the control group after 4 weeks of high-fat fed (*p* < 0.05) (Figure 1). Administration of *L. rhamnosus* GL68 to HFD-induced rats significantly attenuated the rise in body mass, Lee’s index, and liver index, with values decreased by 22.01, 17.71, and 19.35%, respectively (*p* < 0.05). The daily food intake of each rat in the control group, the HFD group, and the *L. rhamnosus* GL68 groups was, respectively, 26.6 ± 2.5 g, 25.3 ± 1.9 g, and 26.1 g ± 2.8 g; there was no significant difference in daily food intake among these groups (*p* > 0.05). The similar results indicated that *L. plantarum* S9 remarkably decreased the mass gain, Lee’s index, and liver index in HFD-induced MS rats (Zhao et al., 2022). Rashid et al. (2025) reported that *Enterococcus durans* NPL 1334 significantly reduced the body mass gain of diet-induced hypercholesterolemic rats (*p* < 0.05). The results of this study indicated that the *L. rhamnosus* GL68 intervention could reduce fat accumulation in the HFD group.

### 3.7 Effect of *L. rhamnosus* GL68 on serum lipid profiles and serum ALT and AST levels in HFD-induced rats

**Figure 2:**
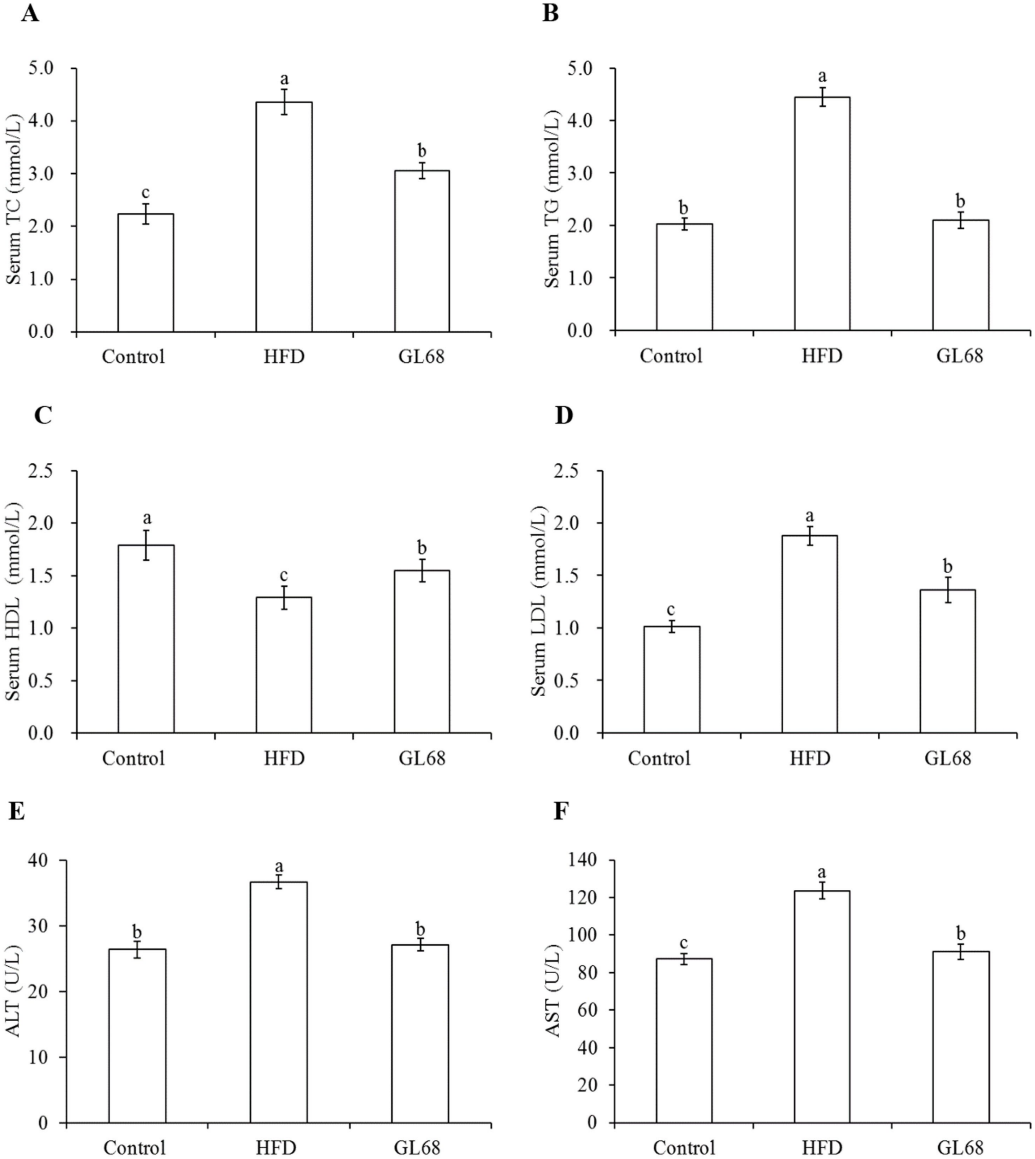
Effect of *L. rhamnosus* GL68 on serum TC **(A)**, TG **(B)**, LDL-C **(C)**, HDL-C **(D)**, ALT **(E)**, and AST **(F)** levels in HFD-induced SD rats.

Serum TC, TG, and LDL-C values in HFD rats significantly increased, while HDL-C value reduced in comparison with rats receiving a normal diet (*p <* 0.05) (Figures 2A–D). Administering *L. rhamnosus* GL68 to the HFD group reduced TC, TG, and LDL-C values by 29.88, 52.89, and 27.51% (*p* < 0.05), and upregulated HDL-C value by 20.03%, respectively (*p* < 0.05). In previous studies, administering *L. fermentum* M1-16 to the HFD group downregulated serum TC and LDL-C values of mice by 12.5 and 17.3% (Xie et al., 2011), and *L. plantarum* E680 decreased TC and LDL-C values in serum by 10.71 and 16.47% (Zheng et al., 2020). Additionally, the results suggested that *L. plantarum E680* had no beneficial effect on serum TC and HDL-C levels in the HFD group (Zheng et al., 2020). Similarly, in another study using a high-fat, high-cholesterol diet model, 4 weeks of administration of the probiotic strain *Lacticaseibacillus casei* ATCC 393 did not result in significant changes in serum TC, HDL-C, or LDL-C levels (Cavdar et al., 2025). In this study, administering *L. rhamnosus* GL68 to HFD rats significantly downregulated TC, TG, and LDL-C values, and upregulated HDL-C values in serum (*p* < 0.05). These results suggested that the probiotics may ameliorate the associated lipid metabolic disorders in a strain-specific manner. A hypothetical mechanism explains the increase in serum HDL-C following probiotic treatment: a reduction in TG levels may elevate serum HDL-C values through an indirect pathway (Zhao et al., 2022).

The levels of serum ALT and AST in the HFD group were significantly higher than those in the control group (*p* < 0.05) (Figures 2E,F). Administering *L. rhamnosus* GL68 to the HFD group significantly downregulated serum ALT and AST levels by 26.10 and 26.27%, respectively (*p* < 0.05). Furthermore, the ALT value in the *L. rhamnosus* GL68 group almost decreased to the level of the control group (*p*>0.05). ALT and AST values are critical and susceptible indicators of liver function, which are generally applied to ascertain the degree of liver injury (Liang et al., 2019). In the HFD-induced mice, elevated ALT and AST levels were also closely associated with liver damage (Jung et al., 2016). Supplementation with *L. rhamnosus* GL68 significantly reduced ALT and AST values in comparison with the HFD group without probiotic supplementation. Several reports have indicated that supplementation with probiotics can modulate metabolic syndrome and hepatic damage caused by high-fat chow (Khanna et al., 2020; Li et al., 2020; Zhao et al., 2022).

### 3.8 Effect of *L. rhamnosus* GL68 on liver MDA, SOD, and GSH-Px in HFD-induced rats

**Figure 3:**
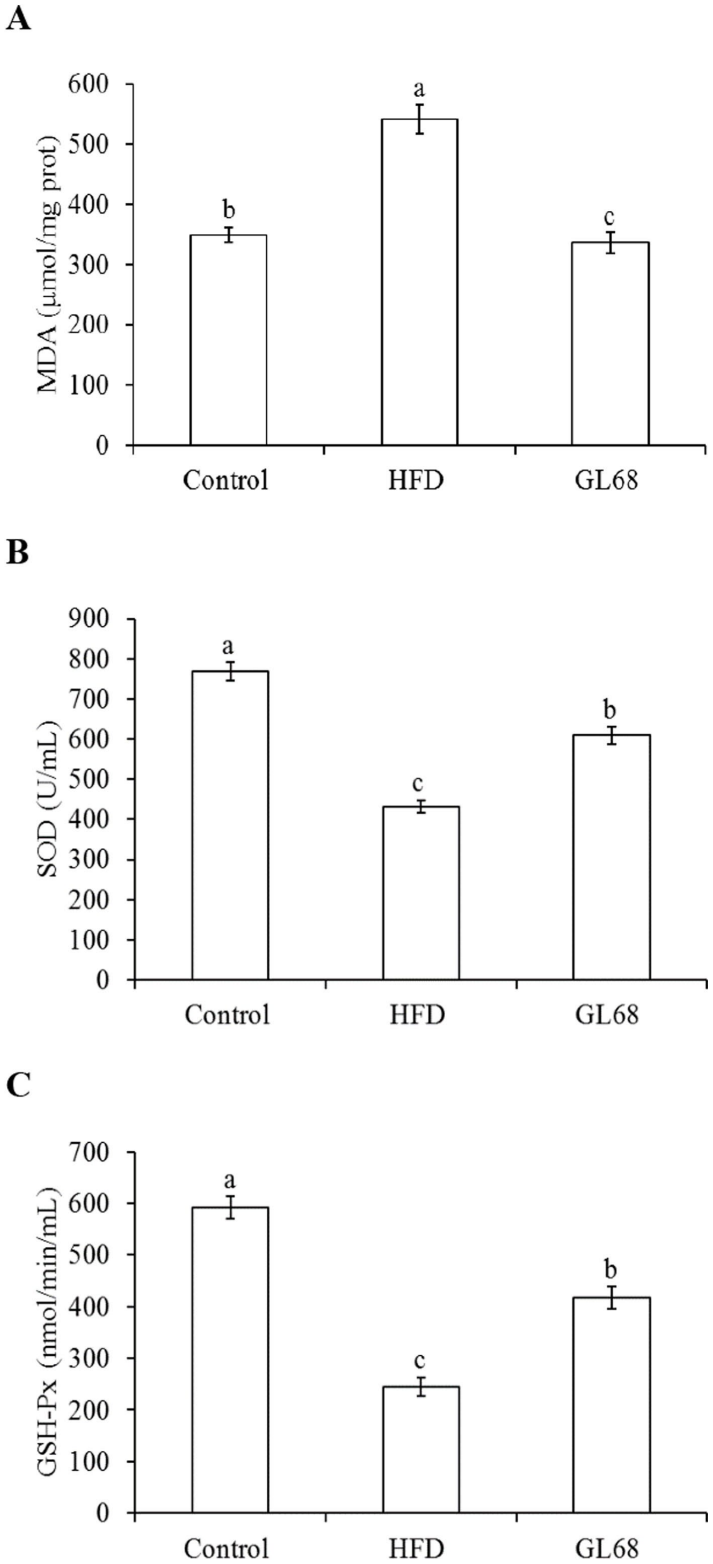
Effect of *L. rhamnosus* GL68 on MDA **(A)**, SOD **(B)**, and GSH-Px **(C)** levels in HFD-induced SD rats.

As shown in Figure 3A, the liver MDA levels in HFD-induced rats were significantly higher than those in rats receiving common chow (*p* < 0.05). Moreover, administering *L. rhamnosus* GL68 significantly decreased liver MDA values in HFD-induced rats (*p* < 0.05). After supplementation with *L. rhamnosus* GL68 for 4 weeks, liver MDA levels in HFD-induced rats decreased by 37.82%, and did not significantly differ from the control group (*p*>0.05). As shown in Figures 3B, C, the levels of SOD and GSH-Px in the livers of SD rats fed a high-fat diet significantly decreased in comparison with the control group (*p* < 0.05). Administering *L. rhamnosus* GL68 to HFD rats significantly upregulated the liver SOD and GSH-Px values by 41.39 and 71.02%, respectively (*p* < 0.05).

In the field of biomedicine, MDA levels in tissue homogenates or blood plasma are widely used to evaluate oxidative stress, with elevated MDA levels serving as a marker of oxidative pressure (Armas-Padilla et al., 2007). Oxygen in the body can form and accumulate the activated oxygen substances, such as superoxide radicals and hydrogen peroxide, thus causing cell injury and affecting normal physiological functions. SOD and GSH-Px are both crucial antioxidants in the body for cleaning up these reactive oxygen species (Zolotukhin et al., 2018). Results in this study indicated that administering *L. rhamnosus* GL68 downregulated the liver tissue’s MDA levels while upregulated the liver tissue’s SOD and GSH-Px in rats receiving high-fat chow (*p* < 0.05). These results thus suggested that *L. rhamnosus* GL68 might protect against liver oxidative damage induced by high-fat chow. Previous studies have indicated that the antioxidant activity of probiotics exhibited high strain-related variability (Amaretti et al., 2013). Moreover, several reports indicated that exogenously administering *B. longum* and *L. acidophilus* also exhibited significant antioxidant activity *in vivo* (Lin and Chang, 2000). Research on the antioxidative effects of intestinal bacteria has shown that the *in vivo* antioxidant effects of probiotic supplements may act indirectly by modulating the microbiota (Forsyth et al., 2009; Nardone et al., 2010). During the process of fat metabolism, the oxidation and decomposition of fats produce large amounts of activated oxygen substances. High-fat chow increases body fat content, and the buildup of peroxides can cause cellular damage, which may further result in cellular dysfunction and diseases. In this study, *L. rhamnosus* GL68 showed both antioxidant capacity *in vitro* and *in vivo*. This antioxidant effect within the body could be obtained through the antioxidant properties of probiotics, which can eliminate free radicals generated by fat oxidation in the intestinal tract and regulate the gut microbiota.

### 3.9 Effect of *L. rhamnosus* GL68 on pathological changes in the liver and pancreas of HFD-induced rats

**Figure 4:**
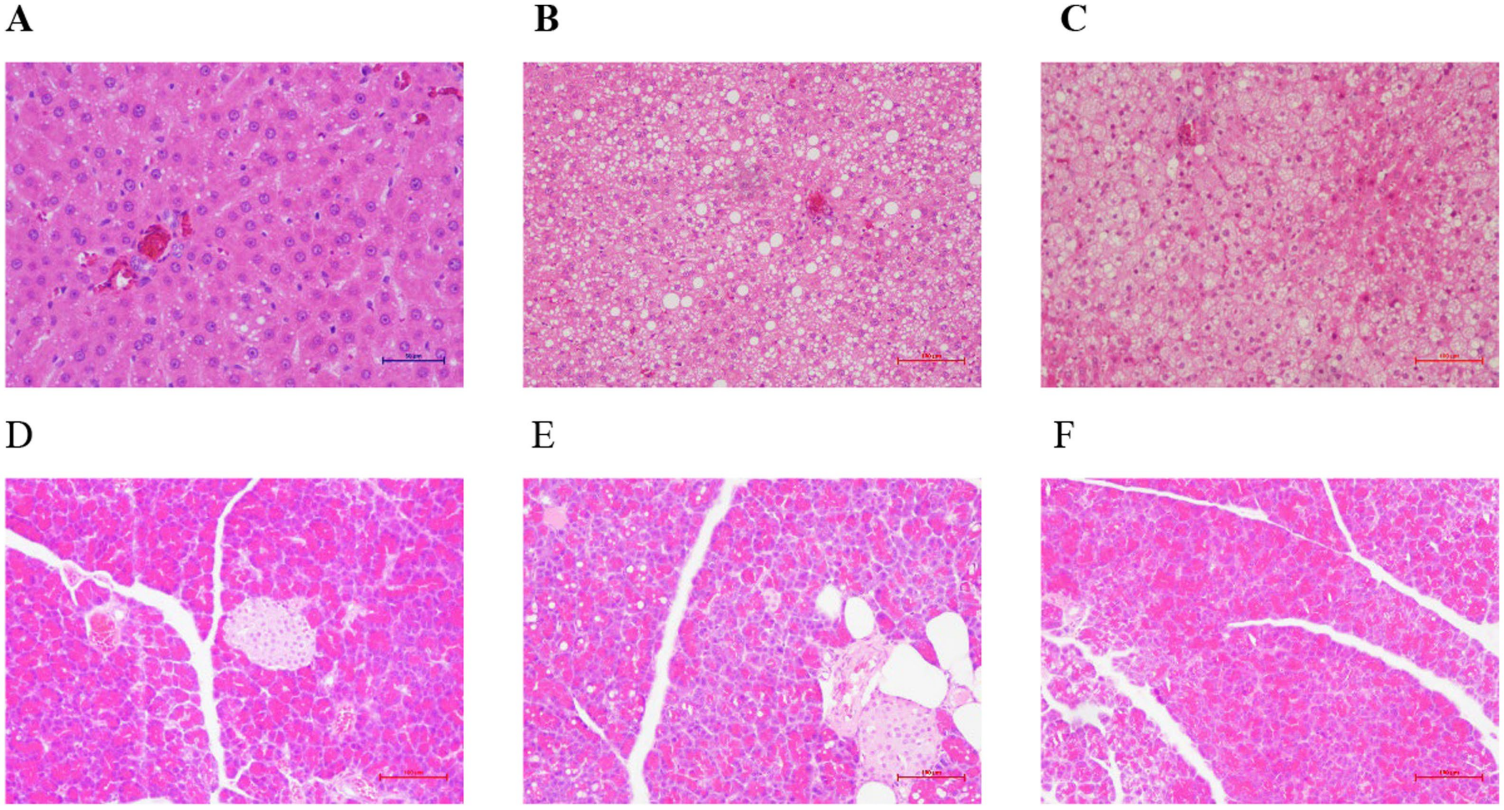
Photomicrographs of stained sections of liver of the control group **(A)**, HFD-induced group **(B)**, *L. rhamnosus* GL68 treatment group **(C)**, pancreas of the control group **(D)**, HFD-induced group **(E)**, and *L. rhamnosus* GL68 treatment group **(F)**.

As shown in Figures 4A–C, the liver of the HFD group displayed an inflammatory response and vacuolar degeneration at grade III in comparison with the rats receiving a normal diet. Administration of *L. rhamnosus* GL68 could significantly ameliorate hepatic inflammation and ballooning degeneration caused by a high-fat diet, with its efficacy approaching that of those receiving a normal diet. As shown in Figures 4D–F, the acinar epithelia in the pancreas of high-fat diet-induced rats exhibited vacuolar degeneration, with symptoms of glandular atrophy and vesicular degeneration. The pancreatic histological sections of rats administered with *L. rhamnosus* GL68 and those fed a common diet were normal, with no vacuoles in the acinar epithelial cells and no symptoms of glandular atrophy or vesicular degeneration. The liver and pancreas are both vital organs for digestion and metabolism. A high-fat diet could exert a significantly damaging influence on the liver and pancreas, primarily through mechanisms such as increasing liver and pancreatic burden, and inducing inflammation and oxidative stress (Zhao et al., 2022; Chen et al., 2025). The result in this study was similar to prior findings that probiotic *Bifidobacterium longum* subsp. *longum* B-53 therapy reduced damage to liver and pancreatic tissues in HFD rats (Chen et al., 2025). The protective effect of *L. rhamnosus* GL68 on the liver and pancreas may be positively correlated with its cholesterol-lowering and antioxidant abilities *in vitro*.

### 3.10 Effect of *L. rhamnosus* GL68 on genus-level relative abundance of intestinal microflora and SCFA of HFD-induced rats

**Figure 5:**
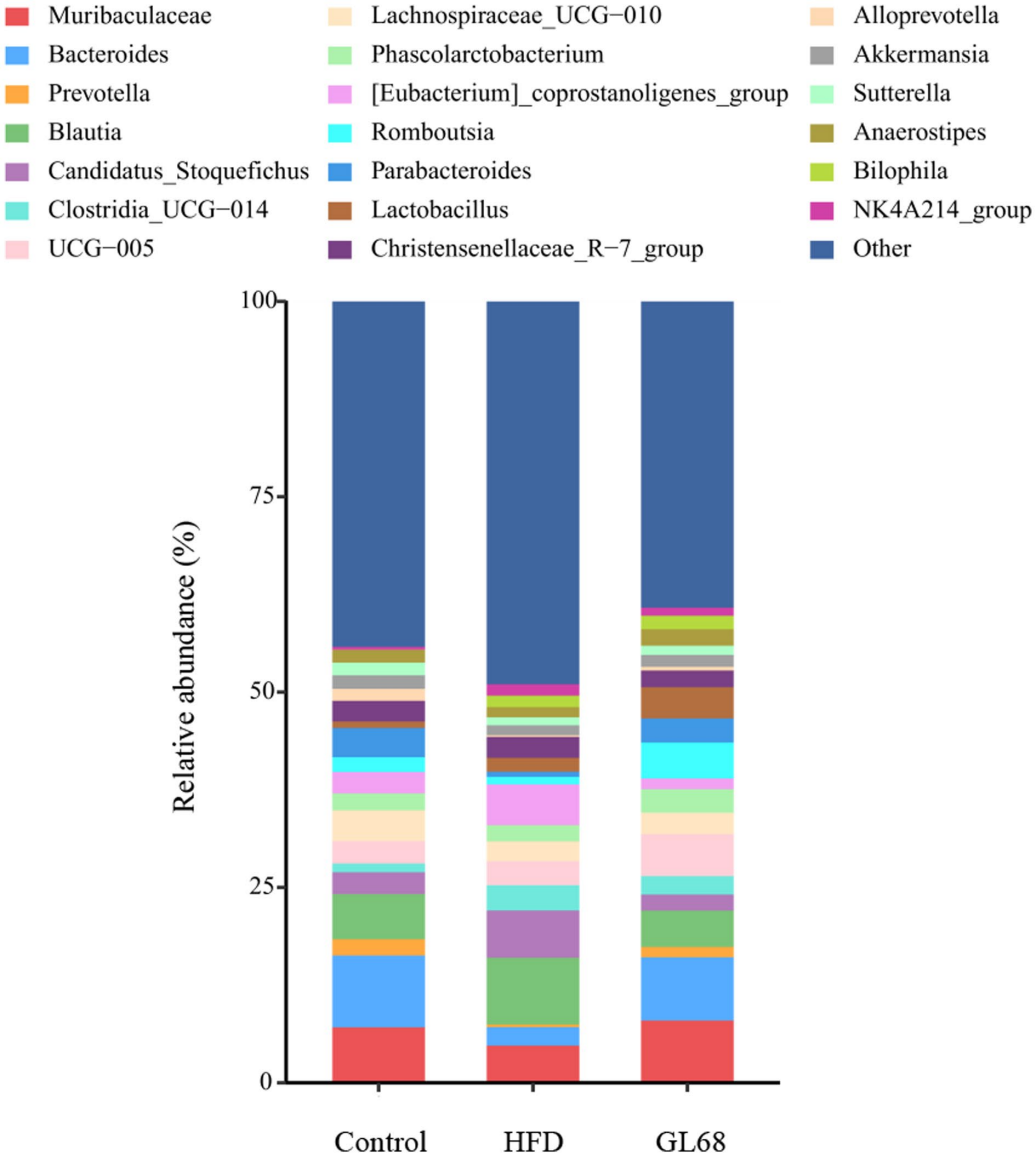
Relative taxonomic abundance at the genus level of gut microbiota in the control group, HFD-induced group, and *L. rhamnosus* GL68 treatment group.

As shown in Figure 5, there were significant differences in the comparative taxonomic abundance of intestinal microflora at the genus level among the control group, the HFD group, and the *L. rhamnosus* GL68 treatment group. It is remarkable that the gut microbiota composition at the genus level in the *L. rhamnosus* GL68 treatment group significantly differed from the high-fat diet-induced group. In comparison with the rats receiving high-fat chow, supplementation with *L. rhamnosus* GL68 significantly raised the gut microbiota abundances at the genus level of *Muribaculaceae*, *Bacteroides*, *Lactobacillus*, *Phascolarctobacterium*, and *Akkermansia*. *Muribaculaceae* increased from 4.73 to 8.19%, *Bacteroides* increased from 2.09 to 8.36%, *Lactobacillus* increased from 1.70 to 3.88%, *Phascolarctobacterium* increased from 2.05 to 2.69%, and *Akkermansia* increased from 0.85 to 1.36%. In comparison with the HFD group, supplementation with *L. rhamnosus* GL68 significantly reduced the gut microbiota abundances at the genus level of *Lachnospiraceae*_NK4A136 and *Candidatus*_*stoquefichus*. *Lachnospiraceae*_NK4A136 decreased from 1.25 to 0.73%, and *Candidatus*_*stoquefichus* decreased from 6.10 to 1.99%. The intestinal microflora is a crucial regulatory element for intestinal homeostasis and host metabolism (Yang and Cong, 2021). Chen et al. (2025) reported that *B. longum* subsp. *longum* B-53 significantly raised the composition of *Muribaculaceae*, *Bacteroides*, *Lactobacillus*, and *Akkermansia* in the intestinal microflora in comparison with the HFD group. Gai et al. (2023) reported that administering *Bifidobacterium* BL21 and *L. acidophilus* LRa05 to type 2 diabetic mice receiving a high-fat diet in combination with streptozotocin could enrich the intestinal microflora, including *Akkermansia*, *Bifidobacterium*, *Lactobacillus*, and *Limosilactobacillus*. Li et al. (2025) indicated that *L. plantarum* BD7807 could restore the abundance of *Muribaculaceae*, *Bacteroidetes*, and *Lactobacilli* in the intestinal tract of mice receiving high-fat chow, while reducing the abundance of the *Lachnospiraceae*_NK4A136_group. Moreover, Li et al. (2025) suggested that *Lachnospiraceae* was positively correlated with undesirable indexes such as TC and TG caused by a high-fat diet. Moreover, *Muribaculaceae* may be beneficial to the production of SCFA from polysaccharoses, thereby exerting various helpful influences (Al-Bulish et al., 2022; Li et al., 2025). Clinical research results indicate that *Lactobacillus* and *Phascolarctobacterium* can reduce intestinal permeability in obese and overweight individuals and enhance intestinal barrier function (DiMattia et al., 2024). Overall, the intervention with *L. rhamnosus* GL68 was effective in regulating the gut microbiotic composition at the genus level in HFD rats.

As shown in Table 4, the levels of three SCFA in the feces of the rats receiving high-fat chow significantly reduced in comparison with rats receiving normal chow (*p* < 0.05). However, after intervention with *L. rhamnosus* GL68, the levels of these SCFAs significantly increased and even exceeded those in rats receiving common chow (*p* < 0.05). Administering *L. rhamnosus* GL68 remarkably raised the content of acetate, propionate, and butyrate in HFD rats’ feces from 321 ± 9 μg/g, 226 ± 6 μg/g, and 295 ± 12 μg/g to 560 ± 13 μg/g, 608 ± 9 μg/g, and 853 ± 21 μg/g. It increased by 74.45, 169.03, and 189.15%, respectively. Compared with the HFD group, the total SCFA content in feces of the *L. rhamnosus* GL68 treatment group increased from 842 ± 27 μg/g to 2021 ± 42 μg/g, and increased by 140.02%. SCFAs are vital products of metabolism produced by gut microbes through the metabolism of non-digestible carbohydrates (Mann et al., 2024). The change in microbiota composition may be mainly induced by probiotic supplements, which could promote the production of SCFAs, thereby improving metabolic disorders (Morrison and Preston, 2016). This study indicated that the fecal SCFA content in the HFD group was significantly lower than that of rats receiving a common chow, while *L. rhamnosus* GL68 could involved in the generation of SCFAs. Moreover, this study reported that *L. rhamnosus* GL68 increased the abundances of beneficial gut microbiota, especially *Muribaculaceae*, *Bacteroides*, *Lactobacillus*, and *Phascolarctobacterium*. Previous reports have suggested that probiotics could adjust intestinal microbiotic structure, and enhance the generation of SCFA, thereby improving gut health (Gai et al., 2023; Li et al., 2025). Previous reports also indicated that SCFA could reach the liver through the vena portae and influence SCFA and cholesterol biotransformation by the enterohepatic metabolizing way (Tian et al., 2020; Lee et al., 2025). Therefore, *L. rhamnosus* GL68 may promote host health by improving the abundance of beneficial microorganisms and correlating with the levels of SCFAs in the intestinal tract.

**Table 4:** Content of SCFA in SD rats’ feces.

Samples	Acetate (μg/g)	Propionate (μg/g)	Butyrate (μg/g)	Total SCFAs (μg/g)
Control group	478 ± 10^b^	526 ± 21^b^	555 ± 15^b^	1,559 ± 46^b^
HFD group	321 ± 9^c^	226 ± 6^c^	295 ± 12^c^	842 ± 27^c^
GL68 group	560 ± 13^a^	608 ± 9^a^	853 ± 21^a^	2021 ± 42^a^

^a-c^Letters indicated that means in the same column are significantly different (*p* < 0.05).

## 4 Conclusion

In this study, *L. rhamnosus* GL68, which exhibited cholesterol-lowering, acid and bile salt tolerance, antibacterial, and antioxidant effects *in vitro*, may exert its role in improving lipid profiles, enhancing hepatic oxidase activity, reducing oxidative damage and functional impairment of liver and pancreatic tissue injury in the HFD-induced SD rats, through a combination of mechanisms including the regulation of the intestinal microflora in genus-level, connection with SCFA production, and reduction of cholesterol and fat peroxides during the digestive absorption process.

## Data Availability

16Sr RNA data of L. rhamnosus GL68 has been deposited in NCBI with accession number: PX671531.

## Footnotes

1
http://www.ncbi.nlm.nih.gov/BLAST

2
http://cloud.majorbio.com/
