# Supplementary material for: Probiotic properties of Lactobacillus rhamnosus GL68 with cholesterol-lowering and antioxidant activities in vitro and its effects on SD rats fed with a high-fat diet
Source: Front Microbiol. 2026 Jun 4;17:1830530. doi: 10.3389/fmicb.2026.1830530 (PMC13275431; doi:10.3389/fmicb.2026.1830530)
Supplement: Supplementary file 1 [file Supplementary_file_1.DOCX]

**Supplementation materials**

**1. 16S ribosomal RNA gene, partial sequence of GL68**

CTCAGGATGAACGCTGGCGGCGTGCCTAATACATGCAAGTCGAACGAGTTCTGATTATTGAAAGGTGCTTGCATCTTGATTTAATTTTGAACGAGTGGCGGACGGGTGAGTAACACGTGGGTAACCTGCCCTTAAGTGGGGGATAACATTTGGAAACAGATGCTAATACCGCATAAATCCAAGAACCGCATGGTTCTTGGCTGAAAGATGGCGTAAGCTATCGCTTTTGGATGGACCCGCGGCGTATTAGCTAGTTGGTGAGGTAACGGCTCACCAAGGCAATGATACGTAGCCGAACTGAGAGGTTGATCGGCCACATTGGGACTGAGACACGGCCCAAACTCCTACGGGAGGCAGCAGTAGGGAATCTTCCACAATGGACGCAAGTCTGATGGAGCAACGCCGCGTGAGTGAAGAAGGCTTTCGGGTCGTAAAACTCTGTTGTTGGAGAAGAATGGTCGGCAGAGTAACTGTTGTCGGCGTGACGGTATCCAACCAGAAAGCCACGGCTAACTACGTGCCAGCAGCCGCGGTAATACGTAGGTGGCAAGCGTTATCCGGATTTATTGGGCGTAAAGCGAGCGCAGGCGGTTTTTTAAGTCTGATGTGAAAGCCCTCGGCTTAACCGAGGAAGTGCATCGGAAACTGGGAAACTTGAGTGCAGAAGAGGACAGTGGAACTCCATGTGTAGCGGTGAAATGCGTAGATATATGGAAGAACACCAGTGGCGAAGGCGGCTGTCTGGTCTGTAACTGACGCTGAGGCTCGAAAGCATGGGTAGCGAACAGGATTAGATACCCTGGTAGTCCATGCCGTAAACGATGAATGCTAGGTGTTGGAGGGTTTCCGCCCTTCAGTGCCGCAGCTAACGCATTAAGCATTCCGCCTGGGGAGTACGACCGCAAGGTTGAAACTCAAAGGAATTGACGGGGGCCCGCACAAGCGGTGGAGCATGTGGTTTAATTCGAAGCAACGCGAAGAACCTTACCAGGTCTTGACATCTTTTGATCACCTGAGAGATCAGGTTTCCCCTTCGGGGGCAAAATGACAGGTGGTGCATGGTTGTCGTCAGCTCGTGTCGTGAGATGTTGGGTTAAGTCCCGCAACGAGCGCAACCCTTATGACTAGTTGCCAGCATTTAGTTGGGCACTCTAGTAAGACTGCCGGTGACAAACCGGAGGAAGGTGGGGATGACGTCAAATCATCATGCCCCTTATGACCTGGGCTACACACGTGCTACAATGGATGGTACAACGAGTTGCGAGACCGCGAGGTCAAGCTAATCTCTTAAAGCCATTCTCAGTTCGGACTGTAGGCTGCAACTCGCCTACACGAAGTCGGAATCGCTAGTAATCGCGGATCAGCACGCCGCGGTGAATACGTTCCCGGGCCTTGTACACACCGCCCGTCACACCATGAGAGTTTGTAACACCCGAAGCCGGTGGCGTAACCCTTTTAGGGAGCGAGCCGTCTAAGGTGGGACAAATGATTAGGGTGAAGTC

**2. Agarose gel electrophoresis of GL68**

**
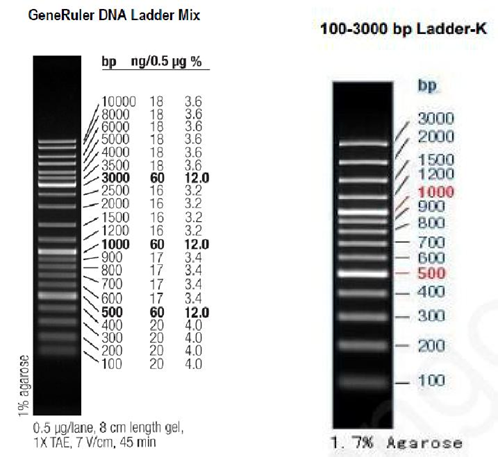

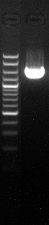
**

Fig. S1 Agarose gel electrophoresis of GL68

**3. Phylogenetic trees drived from 16S rDNA sequence of GL68 isolate**

Fig. S2. Phylogenetic trees drived from 16S rDNA sequence of GL68 isolate. Numbers at the nodes indicated the bootstrap values on neighbor-joining analyses of 1000 resampled data sets. Bar 0.05 represent sequence divergence.
